# The effect of e-cigarettes on cognitive function: a scoping review

**DOI:** 10.1007/s00213-024-06607-8

**Published:** 2024-05-10

**Authors:** Marissa L. Novak, Grace Y. Wang

**Affiliations:** 1 School of Psychology and Wellbeing, University of Southern Queensland, Ipswich, Australia; 2 Centre for Health Research, University of Southern Queensland, Toowoomba, Australia

**Keywords:** Vaping, e-cigarettes, Nicotine, Cognition

## Abstract

**Aim:**

Much research has been conducted on the acute effects of nicotine on human cognitive performance, demonstrating both enhancing and impairing cognitive effects. With the relatively recent introduction of electronic cigarettes (‘e-cigarettes’) as a smoking cessation device, little is known about the cognitive effects of e-cigarettes specifically, either as a nicotine replacement device or in the absence of nicotine. The purpose of this review was to present an overview of evidence from empirical studies on the effect of e-cigarettes on cognitive function.

**Approach:**

Guided by Preferred Reporting Items for Systematic Reviews and Meta-Analyses for Scoping Reviews guidelines (PRISMA-ScR), SCOPUS, PubMed, and EBSCOhost were searched from 2006, the year e-cigarettes were introduced, to June 2023 for relevant papers, along with reference lists checked for additional papers.

**Key findings:**

Seven experimental and four cross-sectional survey studies were identified and included*.* The majority of the studies only include regular and current cigarette smokers and primarily assessed the acute cognitive effect of e-cigarettes relative to nicotine. While the findings primarily suggest either no or positive effect of e-cigarettes on cognition in cigarette smokers, associations between e-cigarettes and cognitive impairments in memory, concentration and decision making were reported in both cigarette smokers and never-smokers.

**Implications and conclusions:**

The acute cognitive effect of e-cigarettes on regular cigarette smokers appears minimal. However, long-term cognitive effect and their effects on never-smokers are unclear. Given that the increased numbers of e-cigarette users are non-smokers and/or adolescents, research with those naïve to nicotine and a developmentally vulnerable adolescent population on its long-term effect is needed.

## Introduction

Electronic cigarettes, also referred to as e-cigarettes, e-cigs, vapes, pens, or mods, were initially developed as a smoking cessation tool by inventor Hon Lik in 2003 (Chadi et al. 2021). E-cigarettes are used for vaping, the practice of inhaling a heated liquid or wax to the point of vaporisation (i.e., producing an aerosol). The e-cigarette liquid (also known as e-liquid, vape fluid, or vape liquid) contains a solvent, primarily vegetable glycerin or propylene glycol, which usually remains chemically unchanged after vaporisation. However, newer devices with higher voltages can alter the chemical composition of solvents and convert them to known carcinogens, such as formaldehyde and acrolein, often at concentrations comparable to those found in tobacco smoke (Crotty Alexander et al. 2015; Parraga and Morissette 2020). The use of e-cigarettes has rapidly increased over the past decade, particularly among adolescents and young adults. E-cigarette sales were predicted to overtake cigarette sales by 2023 (Burrowes et al. 2020; Pasricha and Kochar 2021).

While e-cigarettes are believed to be less harmful than traditional cigarettes given they produce a smaller percentage of dangerous chemicals and toxins and do not produce the tar that is derived from smoking tobacco, the Centres of Disease Control (CDC) warns that ‘less harmful’ does not mean ‘safe’ (Centres for Disease Control and Prevention 2023; Levy et al. 2018; Marques et al. 2021; Skertich et al. 2019). At present, a wide range of health effects of e-cigarettes have been well-researched, such as respiratory disease, cardiopulmonary diseases, gastrointestinal diseases (Chadi et al. 2021; Richmond et al. 2018), but very little is known about the long-term effects of e-cigarettes and their association with cognitive function. As such, some extrapolations may need to be drawn from the results of cigarette smoking research to the effects of e-cigarette use.

Nicotine is the primary constituent of tobacco, and it acts on the cholinergic system through the effects of nicotinic acetylcholine receptors. When acetylcholine binds to nicotinic receptors, the brain’s reward system is activated and dopamine is released (Durand-de Cuttoli et al. 2018). Evidence into the short-term effects of nicotine demonstrate enhanced cognitive effect, particularly in the domains of attention, working memory, and executive function (Swan and Lessov-Schlaggar 2007; Wang et al. 2023). The cognitive enhancement attributed to nicotine is thought to stem from its ability to stabilize mood, achieved by diminishing anxiety levels and enhancing attention (Waters and Sutton 2000). However, amongst smokers, it can be challenging to separate the cognitive-enhancing effects of nicotine from the withdrawal-reversing effects of nicotine. Nicotine withdrawal after abstaining from nicotine has been associated with impairments in cognitive function, including sustained attention, working memory, and response inhibition in both animal and human models (Ashare et al. 2014; Wesnes et al. 2013), with some age-related differences showing impairments might be more pronounced in younger smokers (Falcone et al. 2014).

Contradictory findings have also been documented. Cognitive deficits, such as memory and executive function, has been observed in chronic cigarette smokers (Richards et al. 2003), suggesting an increased risk for late life cognitive impairments associated with chronic smoking. It is argued that in addition to nicotine, tobacco smoke contains chemicals, heavy metals, and free radicals, many of which are associated with brain toxicity and preclinical brain changes (Swan and Lessov-Schlaggar 2007). Evidence shows an association between cigarettes and oxidative stress, which generates excessive reactive oxygen species resulting in memory impairment and cognitive decline (Tobore 2019).

Given these mixed findings, this review aims to present an overview of evidence from empirical studies on the effect of e-cigarettes on human cognitive function. To achieve the goals of this review, three research questions were proposed as guidance, including:

1. What does the evidence tell us about the effect of e-cigarette use on cognitive functioning?
2. Does a history of tobacco use influence the cognitive effects of e-cigarettes?
3. Are there differences in the acute and long-term effects of e-cigarette use?

## Method

### Literature search strategy

This scoping review followed the Preferred Reporting Items for Systematic Reviews and Meta-Analyses for Scoping Reviews (PRISMA-ScR) (Moher et al. 2009). Three databases were searched on 29 June 2023: SCOPUS, PubMed, and EBSCOhost. Boolean search terms were identified for each key construct and used to search the ‘*Titles’, ‘Abstracts’* and *‘Keywords’* (EBSCOhost and SCOPUS), and ‘*Title/Abstract’* (PubMed) of articles to produce a selection of related studies for review (Table 1). Reference lists of articles included in the review were also searched for relevant studies that met the inclusion criteria for the review.

**Table 1:** Boolean search string for key terms

Construct	Boolean search string
Vaping	Vaping OR vape* OR e-cigarette* OR electronic cigarette*
Cognition	Cognition OR cognitive OR attention OR memory OR learn*

### Inclusion criteria

i) publication date of the study ranged from 2006–2023. This date range was chosen to review all available studies since the introduction of e-cigarettes;
ii) the studies were empirical, peer-reviewed, and published in academic journals;
iii) published in English language;
iv) reported outcomes of cognitive functioning associated with e-cigarette use, such as attention, memory, and learning; and
v) participants needed to be non-clinical, healthy populations (i.e., the study does not focus on a specific group of clinical participants, such as patients with asthma).

### Exclusion criteria

i) cognition was not directly assessed as an outcome measure;
ii) reviews, editorials, commentaries, books, or dissertations; or
iii) results of the studies focused on general substance use, rather than e-cigarette use, specifically.

## Results

### Characteristics of studies

**Fig. 1:**
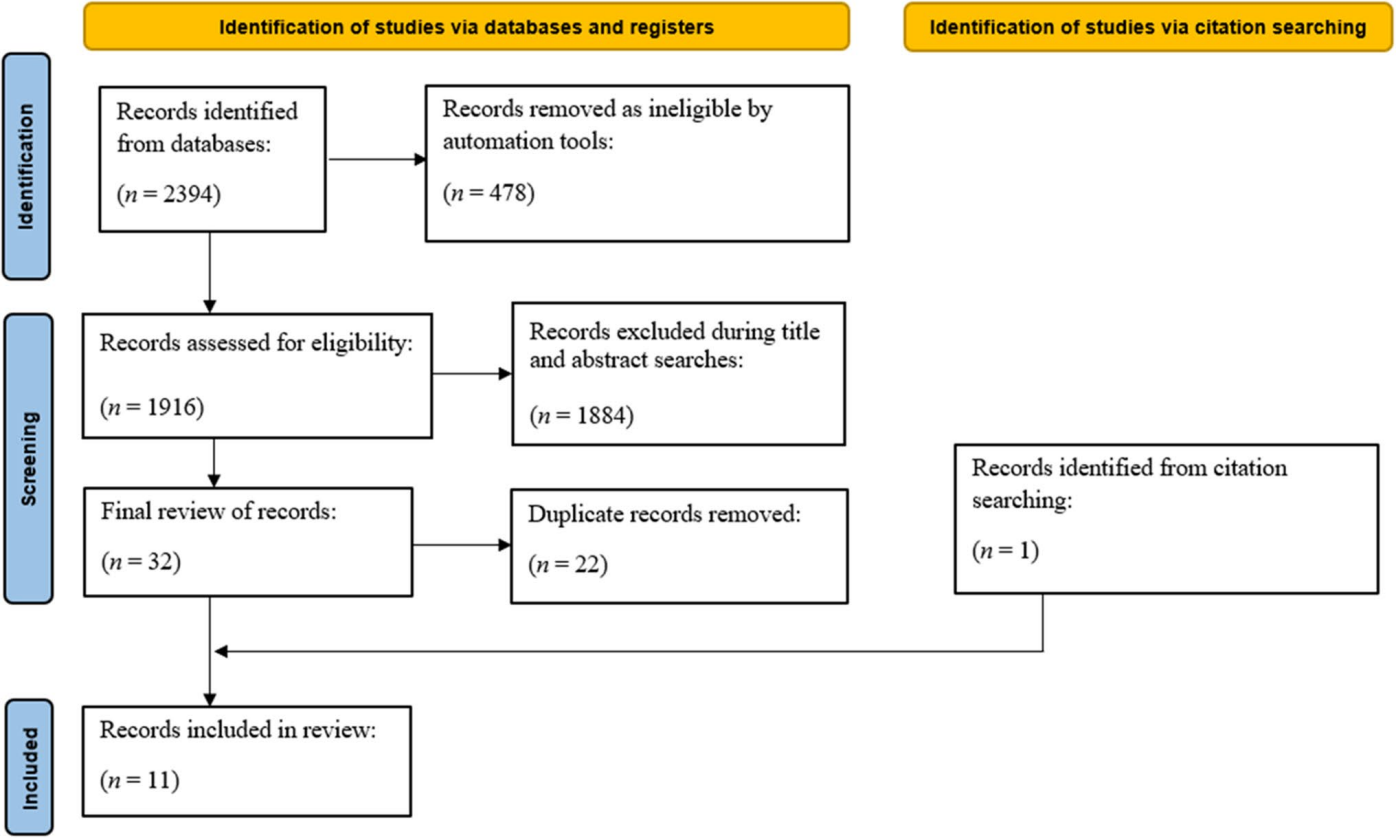
Flow chart of the preferred reporting items for systematic reviews

The search yielded 2394 studies across three databases (EBSCOhost: *n* = 885; SCOPUS: *n* = 911 and PubMed: *n* = 598). Inclusion criteria were applied to initial searches resulting in 1916 eligible records with 478 records excluded for not meeting inclusion criteria. Initial title and abstract searches were conducted, excluding 1884 records. The remaining studies (*n* = 32) included 22 duplicates, which were removed, leaving ten studies suitable for reference list searches. One additional record was identified that met all inclusion criteria, resulting in 11 studies included in the final scoping review, as shown in Fig. 1.

An overview of the characteristics of these studies are shown in Table 2 (experimental studies) and Table 3 (self-report survey designs), which outlines the citation information, country of data collection, type of study design, experimental conditions or survey information, measures of cognition, sample size and participant details, and study findings. More than half of the studies (*n* = 6) were published in the USA (Hobkirk et al. 2018; MacLean et al. 2021; Palmer and Brandon 2019; Wade et al. 2022; Xie et al. 2020a, b), with two studies each being published in England (Dawkins et al. 2013, 2012), Korea (Kim et al. 2022a, b) and one in Italy (Caponnetto et al. 2017).

**Table 2:** Characteristics of the experimental studies included in the scoping review

Author and date of publication	Country	Design/Data collected	Experimental conditions/ Survey information	Measures of cognition	Participants	Findings
Caponnetto et al. 2017	Italy	Experiment (randomised cross-over trial)	Randomly allocated to one of five experimental conditions: (1) first generation e-cigarette (24 mg nicotine) (2) second generation e-cigarette (24 mg nicotine) (3) second generation e-cigarette (0 mg nicotine (4) second generation personal vaporiser (24 mg nicotine) (5) participants own preferred cigarette	Attention: Continuous Performance Test – AX version Executive Function: Wisconsin Card Sorting Test Working Memory: N-BACK	Regular cigarette smokers (*N* = 34, M_age_ = 34.8, 20 males)	No significant cognitive effect
Dawkins et al. 2012	England	Experiment (independent design)	Randomly allocated to one of three experimental conditions: (1) e-cigarette (18 mg nicotine) (2) e-cigarette (0 mg nicotine) (3) e-cigarette held, but not inhaled	Attention/Speed of Processing: Letter Cancellation Task Working Memory: Brown-Peterson Memory Task	Regular cigarette smokers and e-cigarette ‘never users’ (*N* = 60, M_age_ = 28.8, 31 males)	Improvements in working memory from the ‘just hold’ condition
Dawkins et al. 2013	England	Experiment (within-subject design)	Randomly allocated test order: (1) e-cigarette (18 mg nicotine) (2) e-cigarette (0 mg nicotine—placebo)	Prospective Memory: Cambridge Prospective Memory Test	Regular cigarette smokers (*N* = 20, M_age_ = 31.9, 7 males)	Improvements in prospective memory
Hobkirk et al. 2018	USA	Experiment (within-subject design)	Two time conditions: Time 1: Abstinence from e-cigarette use Time 2: After 10 min of puffs (1 puff every 20 s for 30 puffs)	Self-reported difficulty in concentration visual analogue scale from 0 (not at all) to 100 (very much)	Current e-cigarette users (*N* = 9, M_age_ = 35.1, 4 males)	No significant cognitive effect
Kim et al. 2022a	Korea	Experiment (within-subject design)	2 conditions: (1) e-cigarette (16 mg nicotine) (2) participants own preferred cigarette	Working memory: 3-BACK Alphabet/Digit recognition task	Regular cigarette smokers (*N* = 22, *M*_*age*_ = 24.9, 22 males)	Impairments in working memory after e-cigarette use
MacLean et al. 2021	USA	Experiment Observational study	3 conditions: (1) 2% menthol flavour (2) 2% green apple flavour (3) 2% green apple + 2% menthol flavour Each participant then receives the following intravenous nicotine at three different test sessions (1 h apart): (1) 0 mg nicotine (2) 0.25 mg/70 kg body weight (3) 0.5 mg/70 kg body weight	The Automated Neuropsychological Assessment Metrics: Continuous Performance Task (Sustained Attention and Working Memory), Mathematical Processing Task (Attention and Working Memory) and Stroop Test (Cognitive Control)	Regular cigarette smokers (*N* = 24, *M*_*age*_ = 26.2, 15 males)	No significant cognitive effect
Palmer and Brandon 2019	USA	Experiment (analysis of secondary data from previous independent design experiment)	Randomly allocated to one of four experimental conditions: (1) e-cigarette (12 mg nicotine) and told e-cigarettes contained nicotine (2) e-cigarette (0 mg nicotine) and told e-cigarettes contained nicotine (3) e-cigarette (12 mg nicotine) and told e-cigarette did not contain nicotine (4) e-cigarette (0 mg nicotine) and told e-cigarette did not contain nicotine	Attention: Rapid Visual Information Processing Task Continuous Performance Test	Regular e-cigarette users with a history of cigarette smoking (*N* = 128, *M*_*age*_ = 36.4, 80 males)	Improvements in sustained attention among females

**Table 3:** Characteristics of the self-report survey studies included in the scoping review

Author and date of publication	Country	Design/Data collected	Experimental conditions/ Survey information	Measures of cognition	Participants	Findings
Kim et al. 2022b	Korea	Self-Report Survey	Korean Community Health Survey using the Behavioural Risk Factor Surveillance System Questionnaire	Subjective Cognitive Decline measured by a single survey question: “During the past 12 months, have you experienced confusion or memory loss that is happening more often or is getting worse?”	Non cigarette smokers (*n* = 104,453, *M*_*age*_ = 62.3, 14,839 males) Past cigarette smokers (*n* = 38,607, *M*_*age*_ = 63.8, 36,247 males) Current smokers of cigarette, e-cigarette, or e-liquid use (*n* = 26,776, *M*_*age*_ = 56.6, 24,217 males) Total *N* = 169,836	No significant cognitive effect
Wade et al. 2022	USA	Cross-Sectional survey with group comparison analysis	3 self-reported conditions: (1) nicotine and tobacco-product ‘never users’ (control) (2) e-cigarette users (3) cigarette users	National Institute of Health toolbox cognition inventory	E-cigarette users (*n* = 43, *M*_*age*_ = 19.42, 18 males) Cigarette users (*n* = 79, *M*_*age*_ = 19.94, 55 males) Healthy controls (*n* = 79, *M*_*age*_ = 18.96, 34 males)	No significant cognitive effect
Xie et al. 2020a	USA	Self-Report Survey	National Youth Tobacco Survey	Cognitive complaints measured by a single survey question: “Because of a physical, mental, or emotional condition, do you have serious difficulty concentrating, remembering, or making decisions (DCRMD)?”	Cigarette-only users (*n* = 861) E-cigarette-only users (*n* = 2386) Dual users (*n* = 2354) ‘never users’ (*n* = 12,934) Total *N* = 18,535 **Note:** participants age ranged from Grade 6–12, mean age not specified	Significantly higher odds of self-reported DCRMD amongst e-cigarette-only users compared to ‘never users’. This risk was higher compared to cigarette-only smokers, but lower than dual users
Xie et al. 2020b	USA	Self-Report Survey	Behavioural Risk Factor Surveillance System Questionnaire	Cognitive complaints measured by a single survey question: “Because of a physical, mental, or emotional condition, do you have serious difficulty concentrating, remembering, or making decisions?”	‘Never users’ Dual users Current cigarette-only users Current e-cigarette users (split into ex-smokers and never-smokers) Total *N* = 886,603 **Note:** numbers of participants, mean ages in each category and genders in each category were not specified in the study	E-cigarette users reported significantly higher associations with subjective cognitive complaints compared to ‘never users’, with similar results amongst e-cigarette users who were ex-smokers and those who were never smokers. Risk of cognitive impairments amongst exclusive e-cigarette users was higher than exclusive cigarette smokers, but less than dual users

Seven studies (sample sizes ranging from *n* = 9 to *n* = 128) were experimental with participants randomly allocated to experimental conditions, such as different types of e-cigarette devices or different concentrations of nicotine, to determine effect on a measure of cognition (Caponnetto et al. 2017; Dawkins et al. 2013; Dawkins et al. 2012; Hobkirk et al. 2018; Kim et al. 2022a; MacLean et al. 2021; Palmer and Brandon 2019). Four studies adopted cross-sectional survey designs (Kim et al. 2022b, b; Wade et al. 2022; Xie et al. 2020a, b). Among these, three utilised large-scale nationally representative self-report surveys, with sample sizes ranging from *n* = 18,535 to *n* = 886,603 (Kim et al. 2022b; Xie et al. 2020a, b). However, within these surveys, cognition was only measured by a single question (e.g., “Do you have serious difficulty concentrating, remembering, or making decisions?”; Xie et al., 2022). In another smaller survey, participants (*N* = 203) were divided into three groups based on their self-reported history of e-cigarette or cigarette use (e.g., ‘never users’, ‘e-cigarette-only users’, and ‘cigarette-only users’), with group differences in self-reported cognitive function compared (Wade et al. 2022).

Of the studies included in this review, two studies included adolescent and young adult participants (Wade et al. 2022; Xie et al. 2020a), while the rest recruited adult participants only (Caponnetto et al. 2017; Hobkirk et al. 2018; MacLean et al. 2021; Dawkins et al. 2013; Kim et al. 2022a, b; Xie et al. 2020b). The majority of studies (*n* = 6) included participants who were either current smokers only (Caponnetto et al. 2017; MacLean et al. 2021; Dawkins et al. 2012; Dawkins et al. 2013; Kim et al. 2022a), or e-cigarette users with a history of cigarette smoking (Palmer and Brandon 2019). Another study included only participants with a history of e-cigarettes and vaping e-liquid containing nicotine (Hobkirk et al. 2018). The remaining four studies grouped participants according to user status, such as ‘non-cigarette-smokers’, ‘past smokers’ and ‘current smokers’ (Kim et al. 2022b), or ‘exclusive e-cigarette users’, ‘exclusive cigarette smokers’ and ‘never users’ (Wade et al. 2022; Xie et al. 2020a, b).

### Measures of cognition

Cognition is a very broad construct and incorporates many domains. Experimental studies included in this review primarily focused on domains of memory and attention. Memory was assessed using either computerised cognitive tests, such as the N-BACK Working Memory test [8], the 3-BACK Alphabet/Digit recognition task (Kim et al. 2022a), and the Continuous Performance Task (MacLean et al. 2021), or pen-and-paper tests, such as the Cambridge Prospective Memory Test (Dawkins et al. 2013) or the Brown-Peterson Memory Task (Dawkins et al. 2012). Attention was assessed by either computerised measures, such as the Continuous Performance Test – AX version [8], the Automated Neuropsychological Assessment Metrics (MacLean et al. 2021), and the Rapid Visual Information Processing Task (Palmer and Brandon 2019), or a self-reported rating scale, e.g. difficulty in concentration visual analogue scale from 0/not at all to 100/very much (Hobkirk et al. 2018).

For the survey design studies, a self-reported response to a single question (as part of a larger survey) related to cognitive problems was used in three studies (Kim et al. 2022b; Xie et al. 2020a, b). The remining one utilised the National Institute of Health Neurocognitive Toolbox to measure memory and attention (Wade et al. 2022).

### Cognitive effects of e-cigarettes

When examining the cognitive effects of e-cigarettes across 11 studies, results were inconsistent: three studies each reported either cognitive improvements (Dawkins et al. 2012, 2013; Palmer and Brandon 2019) or cognitive impairments (Kim et al. 2022a; Xie et al. 2020a, b), while five studies reported no significant effect of e-cigarettes on cognition (Caponnetto et al. 2017; Hobkirk et al. 2018; Kim et al. 2022b; MacLean et al. 2021; Wade et al. 2022).

Dawkins et al. (2012) examined the cognitive effects of e-cigarettes with a group of current cigarette smokers who had never used e-cigarettes. They randomly allocated participants to either 18 mg nicotine e-cigarette (nicotine condition), 0 mg nicotine e-cigarettes (placebo condition), or a’just hold’ condition, where participants simply held the e-cigarette but did not inhale (Dawkins et al. 2012). The results showed e-cigarette use led to improved performance in memory recall but had no effect on attention-related visual-spatial information processing (Dawkins et al. 2012). Similarly, Dawkins et al. (2013) examined the effect of e-cigarettes on prospective memory with a sample of regular cigarette smokers who were randomly allocated a test order for two e-cigarette conditions: 18 mg nicotine e-cigarettes and a control of 0 mg nicotine e-cigarettes. They reported that e-cigarettes improved time-based prospective memory but had no effect on event-based prospective memory. Furthermore, Palmer and Brandon (2019) examined the immediate cognitive effects of e-cigarettes by randomly allocating current e-cigarette users with a history of cigarette smoking to one of four experimental conditions: 12 mg nicotine e-cigarette and were told the e-cigarette contained nicotine, 0 mg nicotine e-cigarette and were told the e-cigarette contained nicotine, 12 mg nicotine e-cigarette and were told the e-cigarette did not contain nicotine, and 0 mg nicotine e-cigarette and were told the e-cigarette did not contain nicotine. It was shown that nicotine e-cigarette improved sustained attention, and the improvement was more salient among females (Palmer and Brandon 2019). However, these studies only tested individuals with a history of nicotine use and lacked control groups (i.e., participants who had never used e-cigarettes or cigarettes), which makes it difficult to isolate the actual effect of e-cigarettes from nicotine, and improved cognition might be related to reduced withdrawal effect associated with e-cigarettes use (Dawkins et al. 2013; Palmer and Brandon 2019). Nevertheless, a study compared the self-reported difficulty in concentration in regular nicotine e-cigarette users pre- and post-overnight abstinence, they found no significant effect of e-cigarettes on cognition (Hobkirk et al. 2018).

In contrast to the reported cognitive improvements, three studies reported cognitive impairments related to e-cigarette use. A repeated-measures study examining the cognitive effects of vaping e-cigarettes (containing 16 mg/ml nicotine) on current cigarette smokers (*N* = 22) in relation to regular cigarette use following overnight cessation (about 12 h of smoking abstinence) had opposite findings (Kim et al. 2022a). It was reported that participants performed more poorly in memory task following e-cigarette use compared to using their regular brand cigarette (Kim et al. 2022a). It was argued that regular cigarette smokers were not fully satiated by vaping e-cigarettes, and this led to differences in behavioural measures (Kim et al. 2022a). In line with this, studies that adopted large-scale nationally representative survey reported increased risks of subjective cognitive impairments (Xie et al. 2020a, b). This risk amongst exclusive e-cigarette users appears higher than amongst ‘never users’ or exclusive cigarette users (Xie et al. 2020b). Furthermore, Xie et al. (2020a) reported that adolescent e-cigarette users were at a significantly higher risk of difficulties in concentration, remembering, and making decisions compared to ‘never users’, and that the risk was even higher in adolescents who initiated e-cigarettes at a younger age. However, it should be noted that survey question as a measure of cognition does not identify unknown causes of cognitive difficulties and there are also survey studies that failed to identify significant association between e-cigarette use and cognitive decline (Kim et al. 2022b; Wade et al. 2022).

Overall, it appears that experimental studies that reported acute effects of e-cigarette use suggest cognitive improvements, which may have more to do with the cognitive effects of nicotine, rather than e-cigarettes specifically. Studies reporting significant results from surveys found cognitive impairments of e-cigarette use among participants who have never smoked cigarettes as well as amongst those with a history of cigarette smoking in both adult and adolescent samples.

## Discussion

The purpose of this scoping review was to map the body of extant empirical literature on the cognitive effects of e-cigarettes across groups of tobacco smokers and non-smoker healthy individuals.

### What does the evidence tell us about the effect of e-cigarette use on cognitive functioning?

Despite mixed findings, it appears that acute cognitive effects of e-cigarettes are either positive or minimum, at least when it comes to regular tobacco smokers. This is consistent with well-established research related to nicotine (Jasinska et al. 2014; Wang et al. 2023). However, concerning results suggest cognitive impairments are associated with e-cigarettes, which were primarily in the domains of memory and subjective cognitive decline. Although there was limited research reporting adverse cognitive effect of e-cigarettes, these results should not be underestimated. In particular, these results have been generated from large-scale nationally representative self-report surveys (Xie et al. 2020a, b), which compared ‘e-cigarette-only users’ to ‘never users’ and ‘cigarette-only users’, which made isolating the effects of e-cigarettes from traditional cigarette possible. While both user groups had an increased risk of self-reported cognitive impairments, ‘exclusive e-cigarette users’ were found to be at a higher risk than ‘exclusive cigarette users’, particularly amongst adolescents (Xie et al. 2020a, b). Despite the limitations of self-report measures (e.g., reporting bias), studies have shown that self-report measures are more likely to capture cognitive impairments associated with daily functioning when compared to lab-based neurocognitive measures (Albein-Urios et al. 2018), particularly when the target measure is clear (Cyders & Coskunpinar 2011). As such, these results should not be easily dismissed.

It is important to acknowledge that cognitive domains are multifaceted, and variations in research methods can have significant implications for the results obtained. Different studies may employ diverse measures to assess the same cognitive domain, such as memory. This diversity in measurement tools can lead to variations in findings, as illustrated by the example of four studies purportedly measuring working memory (Caponnetto et al. 2017; Dawkins et al. 2012; Kim et al. 2022a; MacLean et al. 2021). Each study utilized a different cognitive test as their measure of working memory, including the N-BACK, Brown-Peterson Test, 3-BACK, and Automated Neuropsychological Assessment Metrics, respectively, and consequently, the results from these studies may not be directly comparable.

### Does a history of tobacco use influence the cognitive effects of e-cigarettes?

Tolerance to nicotine amongst cigarette smokers may account for the non-significant cognitive effects, as the constituents of e-liquids are comparable to those found in cigarettes (Walley et al. 2019). These confounds may limit the ability to detect cognitive effects of e-cigarettes. Among studies reporting no significant cognitive effect or cognitive improvements, most of these studies measured changes in cognition in participants that were all current cigarette smokers (Caponnetto et al. 2017; Dawkins et al. 2012, 2013; Hobkirk et al. 2018; MacLean et al. 2021; Palmer & Brandon et al., 2019). In contrast, studies comparing ‘exclusive e-cigarette users’ with ‘never users’ and ‘exclusive cigarette users’ found greater risk of self-reported cognitive impairments in ‘exclusive e-cigarette users’ (Xie et al. 2020a, b). As such, the findings from research with exclusive sample of cigarette smokers may be biased.

### Are there differences in the acute and long-term effects of e-cigarette use?

None of studies have specifically addressed differences in acute and long-terms effect of e-cigarette us. Nevertheless, inconsistent findings generated from experiment design and survey results suggest potential disparities between the immediate and prolonged cognitive effects of e-cigarette use. While experiments may reveal acute effects, surveys typically concentrate on long-term impacts. Similar to tobacco (Conti et al. 2019), it appears that e-cigarette consumed acutely could enhance cognitive function, whereas chronic use is associated with cognitive deficiencies. It is worth to note that acute effect of e-cigarettes could be modulated by sex difference as a study reported that improvements in attention following acute consumption of e-cigarettes were only observed in females (Palmer and Brandon 2019).

### Strengths and limitations

This review is the first to our knowledge to provide a synthesis of the literature investigating the effect of e-cigarettes on cognition. Even though only 11 studies were identified to be included in this review, a strength of this review is that studies were published from a range of countries, providing a global perspective on the cognitive effects of e-cigarettes. However, there were some limitations to this review that should be considered. Although the search for relevant studies was systematic, it may not be exhaustive as not all databases were searched. Other databases may provide evidence into the effect of e-cigarettes on different cognitive domains, such as learning or decision making, or may outline further evidence targeting adolescent demographics. Finally, most studies in this review included small sample sizes, were predominantly Caucasian participants and recruited participants who were established cigarette smokers. This limits the ability to generalise results to broader demographics.

### Future research direction

A history of unclear regulations related to e-cigarette product packaging and marketing (e.g., labelling of ingredients and concentrations of compounds) and a thriving black market selling unrestricted e-cigarette products add to the concerns related to the unknown effects of e-cigarettes. At present, many e-cigarette users, particular younger ones, typically are not cigarette smokers or ex-smokers (i.e., are not using e-cigarettes as a means to cut back on cigarette smoking). Either age, previous exposure to nicotine, or both, could modulate the actual effect of e-cigarettes on cognition. Thus, future research should adopt a more holistic approach to studying the effects of vaping, considering not only nicotine's cognitive effects but also the broader context of vaping products and their diverse usage patterns. Developing an understanding of the cognitive effects of e-cigarettes specifically in the absence of nicotine is critical and could assist in identifying risk factors of use, particularly regarding adolescents. As adolescence is a vulnerable stage of neurocognitive development, adolescents are more at risk of suffering long-term negative effects of e-cigarettes than adults. Furthermore, majority of the studies in this review were cross-sectional in design and focused on acute effects of e-cigarettes. Understanding of long-term effect of e-cigarette calls for longitudinal and increased measurement rigor and standardization for cognitive functioning, especially in survey studies. Additionally, further research with a methodological focus on the the actual causal relationship between cognition and e-cigarettes should also be considered.

## Conclusion

As e-cigarettes and vaping have only been recently introduced to mainstream markets, research into their effects is still in its infancy. As such, this review contributes an improved understanding of the cognitive effects of e-cigarettes. From a health standpoint, despite certain evidence indicating a beneficial impact of e-cigarettes on memory and attention, it's crucial not to overlook evidence of their potential negative effects. Developing a comprehensive understanding of the cognitive effects of e-cigarettes and vaping would have important implications for policy development and reform.

## Declarations

No Generative AI and AI-assisted technologies used in the whole writing process.
